# Which one of LDL-C /HDL-C ratio and non-HDL-C can better predict the severity of coronary artery disease in STEMI patients

**DOI:** 10.1186/s12872-022-02760-0

**Published:** 2022-07-17

**Authors:** Po Gao, Xiang Wen, Qiaoyun Ou, Jing Zhang

**Affiliations:** Department of Cardiovascular Medicine, Hefei Hospital Affiliated to Anhui Medical University, Intersection of Guangde Road and Leshui Road, Yaohai District, Hefei, 230000 China

**Keywords:** STEMI, LDL-C/HDL-C, Non-HDL-C, Gensini score

## Abstract

**Background:**

The increase of low-density lipoprotein cholesterol (LDL-C) is widely accepted as an important factor in the occurrence of atherosclerosis. In recent years, the guidelines have recommended non-high density lipoprotein cholesterol (non-HDL-C) as a secondary target for lipid-lowering therapy. But even as research on the relationship between LDL-C/HDL-C and atherosclerosis increases, it is still undetermined which index is most closely related to the severity of acute ST-segment elevation myocardial infarction (STEMI).

**Methods:**

901 patients who received coronary angiography due to chest pain were selected. Among them, 772 patients with STEMI represented the test group, and 129 patients with basically normal coronary angiography represented the control group. Researchers measured fasting blood lipids and other indicators after admission, and determined the severity of coronary artery disease using the Gensini score.

**Results:**

LDL-C/HDL-C and non-HDL-C indexes were statistically different between the two patient groups. In the test group, total cholesterol (TC), triglycerides (TG), LDL-C, high density lipoprotein cholesterol (HDL-C), non-HDL-C, arteriosclerosis index (AI), and LDL-C/HDL-C all correlated with the patients' Gensini score. After applying the stepwise method of multiple linear regression analysis (*R*^2^ = 0.423, *β* = 0.518, *p* < 0.05), LDL-C/HDL-C had the most correlation with the patient's Gensini score. ROC curve analysis suggested that LDL-C/HDL-C can predict whether patients with chest pain are STEMI (*AUC*: 0.880, 95% *Cl*: 0.847–0.912, *p* < 0.05). When cutoff value is 2.15, sensitivity is 0.845, and specificity is 0.202, LDL-C/HDL-C is an effective indicator for predicting whether patients with chest pain have STEMI.

**Conclusion:**

Compared to ratios of non-HDL-C and LDL-C, the LDL-C/HDL-C ratio in patients with STEMI is more correlated with the severity of coronary artery disease. It can better evaluate the severity of coronary artery disease and better predict whether patients with chest pain are STEMI.

**Supplementary Information:**

The online version contains supplementary material available at 10.1186/s12872-022-02760-0.

## Introduction

The formation of atherosclerosis is a slow and complex process. Its pathogenesis, recognized as "Injury Response Theory," [1] can be generally divided into three processes: (1) physical or chemical factors stimulate the arterial intima and blood lipid adheres to the blood vessel wall due to injury; (2) blood lipids are deposited in the injured arterial intima, stimulating further injury of the arterial intima and resulting in further deposition; (3) fat deposition stimulates the proliferation of smooth muscle cells and fibroblasts in the inner wall of the artery and the proliferation continues to develop into fibrous plaques. Excessive lipid deposition can also aggravate the inflammatory reaction by promoting the secretion of inflammatory mediators and aggravating atherosclerosis. STEMI is caused by coronary artery occlusion or thrombosis due to injury, or rupture of coronary artery lipid plaque. Hyperlipidemia management should hold a central pathogenic role among risk factors for coronary heart disease, and control of blood lipids should be the key treatment goal.

The main indicators included in the blood lipid profile are TG, TC, HDL-C, LDL-C, very low-density lipoprotein cholesterol (VLDL-C), etc. Lipid metabolism disorder has always been a contentious issue in the field of coronary heart disease (CHD) research. An increase in both TC and LDL-C is widely accepted as an important factor in the occurrence of atherosclerosis [2]. Later studies have confirmed that TG also causes atherosclerosis, while HDL-C has an anti-atherosclerotic effect [3]. Recently, studies have shown that indicators such as AI, non-HDL-C, and LDL-C/HDL-C are closely related to CHD and other clinical diseases [4]. In particular, there are many studies on non-HDL-C and LDL-C/HDL-C. As of now, the blood lipid guidelines in the United States and Europe still regard LDL-C as the primary goal of blood lipid control [5], while non-HDL-C and LDL-C/HDL-C are regarded as secondary goals. Although many studies have detailed the above two indicators, the indicators still present the outer range of coronary heart disease, and no results clearly show what boundary value of the two indicators would require intervention. The most serious type of coronary heart disease, STEMI has a high mortality rate and poor prognosis. This study explores the above two indicators’ evaluative effect on the severity of coronary artery disease in STEMI patients, both weighing which of the two indicators evaluates severity more effectively, and identifying the boundary value that requires intervention.

## Methods

The records of 901 patients who received coronary angiography due to chest pain in the hospital from September 2014 to September 2020 were retrospectively analyzed. Among them, 772 were STEMI patients, and 129 patients had basically normal coronary angiography. The 2018 "Global Definition of the Fourth Myocardial Infarction" was used as diagnostic criteria; previous history of myocardial infarction, malignant tumors, severe infections, liver and kidney insufficiency, and past history of lipid-lowering drugs were excluded. Patient gender, age, history of diabetes, history of hypertension, smoking habits, height, and weight were recorded. The selected patients underwent a blood test, electrocardiogram, and coronary angiography at the time of admission and a blood lipid test the next morning (including TC, TG, LDL-C, HDL-C, VLDL-C). Gensini score, a classic coronary artery lesion severity scoring system, can comprehensively reflect the degree and extent of coronary artery stenosis. The Gensini score was used to evaluate the severity of coronary lesions. The calculation of the score is as follows: First, the evaluator scores the stenosis according to the degree of coronary artery stenosis: 1 point for ≤ 25% obstruction, 2 points for 26–50% obstruction, 4 points for 51–75% obstruction, 8 points for 76–90% obstruction, 16 points for 91–99% obstruction, and 32 points for total occlusion (100%). Second, the evaluator must determine the coefficient according to the location of coronary stenosis: left main coronary artery (LM) lesion × 5; left anterior descending branch (LAD) lesion, distal segment × 1. middle segment × 1.5, proximal segment × 2.5; diagonal branch lesion, first diagonal branch (D1) × 1, second diagonal branch (D2) × 0.5; left circumflex branch (LCX) lesion, distal segment × 1, proximal segment × 2.5; obtuse marginal branch × 1, posterior descending branches × 1; posterior branches of left ventricular × 0.5. The lesions of right coronary artery (RCA) included proximal, middle, distal and posterior descending branches, each segment × 1. Thirdly, the evaluator must multiply the coronary artery stenosis score by its corresponding coefficient, giving the lesion score. The sum of the lesion scores is the Gensini score. Two physicians recorded detailed statistics on the location of coronary artery lesions and the degree of stenosis in each patient. The degree of coronary artery lesions was scored using the Gensini score. In the case of disagreement, a third physician formed a joint discussion to make a decision. The non-HDL-C calculation method is TC minus HDL-C, and the AI calculation method is TC minus HDL-C then divided by HDL-C.

## Statistical analysis

Non-normal distribution measurement data are represented by *M* (*Q*1, *Q*3). An independent sample *t* test or a Mann–Whitney *U* test is used for comparison of measurement data between two groups. A chi-squares test is used for comparison of count data. The correlation between variables is analyzed by either Pearson linear correlation analysis or logistic regression analysis, and the results are expressed in terms of correlation coefficient *r* or ratio *OR* and 95% confidence interval 95%* Cl*. Multivariate correlation is analyzed by multiple linear regression with variable selection. The introduction level is entered as 0.05 and the elimination level is 0.10. The result is expressed by the determination coefficient *R*^2^ and the standardized regression coefficient *β*. All the above test results are statistically significant, with a bilateral *p* < 0.05 as the difference. All data were analyzed and processed by SPSS 22.0.

## Results

### Baseline clinical characteristics

A total of 901 patients were included in the study, 670 males (74.4%) and 231 females (25.6%). According to the results of their coronary angiography, patients were divided into a test group (772 STEMI patients) and a control group (129 normal patients). Table one summarizes the baseline characteristics and laboratory data of the patients (Table 1).

**Table 1:** Baseline Clinical Characteristics

Variables	Test group (n = 772)	Control group (n = 129)	$\chi$ ^2^*/z*	*p*
age	62.75 (53.00,72.00)	59.37 (50.50,69.00)	− 1.347	0.178
Sex (%)	Male 586 (75.9%)	Male 84 (65.1%)	6.750	0.009
	Female 186 (24.1%)	Female 45 (34.9%)		
Hypertension (%)	442 (57.3%)	59 (45.7%)	6.441	0.040
Diabetes (%)	328 (42.5%)	36 (27.9%)	10.003	0.007
Smoker (%)	442 (57.3%)	46 (35.7%)	20.762	0.000
BMI (kg/m^2^)	23.64 (22.00, 25.00)	22.50(20.00, 24.00)	− 4.508	0.000
ANC (X10^9^)	5.85 (4.5, 6.84)	3.91 (3.09, 4.43)	− 9.243	0.000
TLC (X10^9^)	1.91 (1.57, 2.29)	2.12 (1.53, 2.64)	− 7.570	0.000
RBC (X10^9^)	4.36 (3.94, 4.81)	4.38 (3.94, 4.76)	− 1.400	0.888
TC (mmol/L)	4.38 (3.70, 4.93)	3.67 (3.09, 4.35)	− 8.143	0.000
TG (mmol/L)	1.60 (1.04, 1.87)	1.38 (0.93, 1.66)	− 2.334	0.020
HDL-C (mmol/L)	0.91 (0.76, 1.01)	1.14 (0.94, 1.23)	− 7.770	0.000
LDL-C (mmol/L)	2.60 (2.03, 3.10)	1.83 (1.46, 2.30)	− 9.631	0.000
VLDL (mmol/L)	0.74 (0.49, 0.88)	0.59 (0.37, 0.73)	− 3.211	0.010
LDL-C/HDL-C	2.98 (2.27, 3.50)	1.69 (1.20, 2.11)	− 9.806	0.000
Non-HDL-C (mmol/L)	3.47 (2.80, 4.01)	2.53 (1.93, 3.10)	− 9.020	0.000
AI	4.03 (3.00, 4.73)	2.15 (1.67, 3.08)	− 9.401	0.000

Indexes such as body mass index (BMI), diabetes history, history of hypertension, smoking habits, absolute neutrophil count (ANC), total lymphocyte count (TLC), and TC, TG, HDL-C, LDL-C, VLDL-C, non-HDL-C, AI, and LDL-C/ HDL-C were significantly different in the two groups of patients.

### Correlation between related indexes in blood lipids and Gensini score

**Fig. 1:**
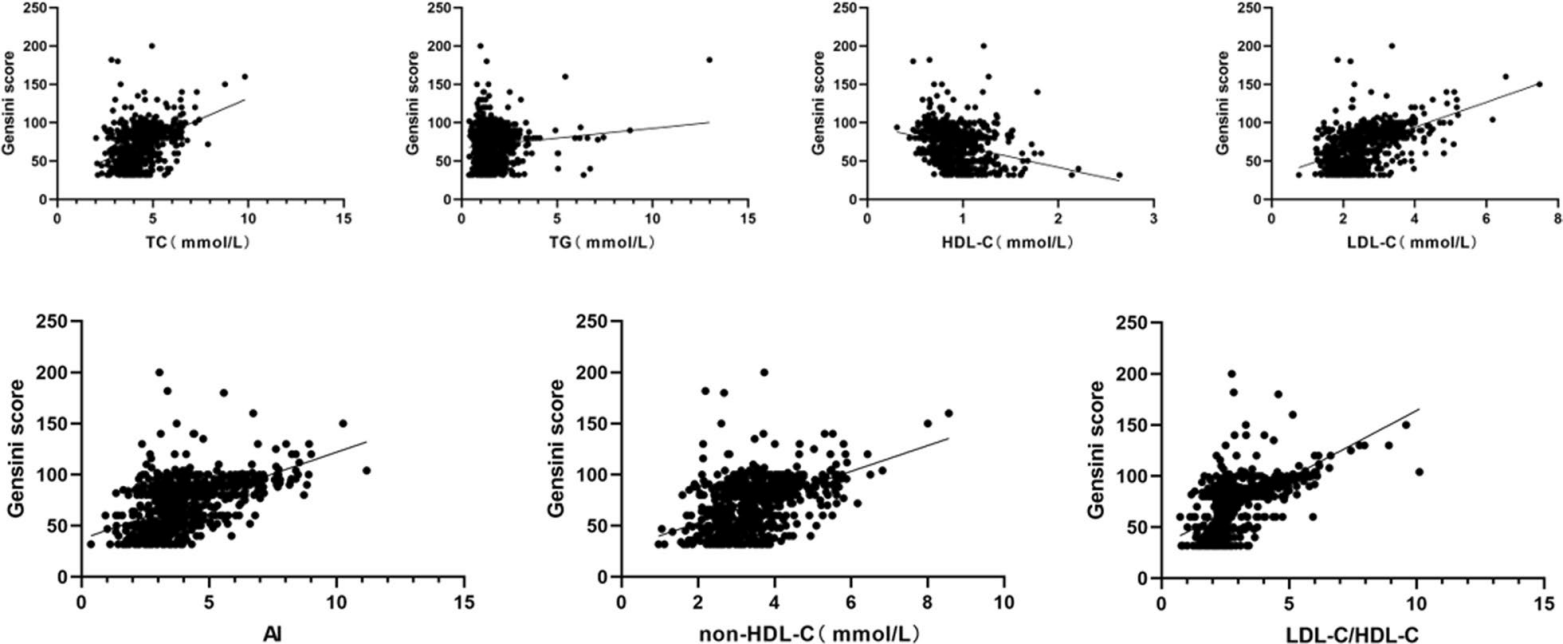
Correlation between indicators in blood lipids and Gensini score

Correlation analysis of TC, TG, HDL-C, LDL-C, VLDL-C, non-HDL-C, AI, LDL-C/HDL-C and Gensini scores of all test groups of patients found that TC, TG, HDL-C, LDL-C, non-HDL-C, AI, and LDL-C/HDL-C are all correlated with Gensini score. Among them, TC, TG, LDL-C, non-HDL-C, AI, and LDL-C/HDL-C are positively correlated with Gensini score. HDL-C was negatively correlated with Gensini score (Table 2 and Fig. 1).

**Table 2:** Correlation between related indexes in blood lipids and Gensini score

Variables	*r*	95%* CL*	*p*
TC (mmol/L)	0.422	9.145, 12.418	0.000
TG (mmol/L)	0.103	0.800, 4.287	0.004
HDL-C (mmol/L)	− 0.260	− 30.400, − 20.092	0.000
LDL-C (mmol/L)	0.527	14.440, 18.157	0.000
VLDL-C (mmol/L)	0.006	− 3.492, 4.152	0.865
Non-HDL-C (mmol/L)	0.490	11.019, 14.193	0.000
AI	0.528	7.524, 9.458	0.000
LDL-C/HDL-C	0.616	12.020, 14.413	0.000

### Multivariate correlation analysis of Gensini scores

In further analysis, the Gensini score was used as the dependent variable, and all the above correlation indicators, along with hypertension, diabetes, and smoking, were used as independent variables to perform multiple linear regression stepwise analysis. LDL-C/HDL-C, non-HDL-C, TG, hypertension, diabetes, and smoking entered the analysis, LDL-C/HDL-C and non-HDL-C still had a positive correlation with Gensini score(*R*^2^ = 0.423, *β* = 0.518, *β* = 0.150, *p* < 0.05). (Table 3).

**Table 3:** Multivariate correlation analysis of Gensini scores

Variables	*SD*	*β*	*t*	*p*
LDL-C/HDL-C	0.756	0.518	14.720	0.000
Non-HDL-C (mmol/L)	0.918	0.150	4.198	0.000
TG (mmol/L)	0.695	0.058	2.076	0.038
Diabetes	1.365	0.115	4.166	0.000
Hypertension	1.385	0.100	3.554	0.000
Smoking	1.361	0.068	2.473	0.014

### The predictive effect of LDL-C, non-HDL-C, and LDL-C/HDL-C on STEMI patients

**Fig. 2:**
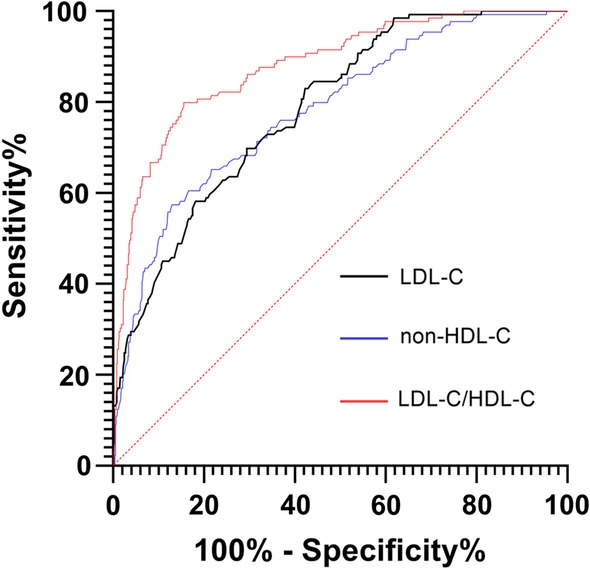
ROC curves of LDL-C, non-HDL-C, and LDL-C/HDL-C

The control group was regarded as a low Gensini score group, while the test group was regarded as a high Gensini score group. LDL-C/HDL-C, non-HDL-C, and LDL-C were used as independent variables to calculate the ROC curve. The ROC curve of LDL-C/HDL-C was *AUC*: 0.880, 95% *Cl*: 0.847–0.912, *p* < 0.05, with a cutoff value pf 2.15, a sensitivity of 0.845, and a specificity of 0.202. The ROC curve of non-HDL-C was *AUG*: 0.782, 95% *Cl*: 0.739–0.826, *p* < 0.05, with a cutoff value of 2.52, a sensitivity of 0.870, and a specificity of 0.426. The ROC curve of LDL-C was *AUG*: 0.787, 95% *Cl*: 0.748–0.826, *p* < 0.05, with a cutoff value of 2.35, a sensitivity of 0.578, and a specificity of 0.829 (Fig. 2). Compared with non-HDL-C and LDL-C, LDL-C/HDL-C can better predict whether patients with chest pain are STEMI.

### LDL-C/HDL-C as an independent risk factor for STEMI

**Fig. 3:**
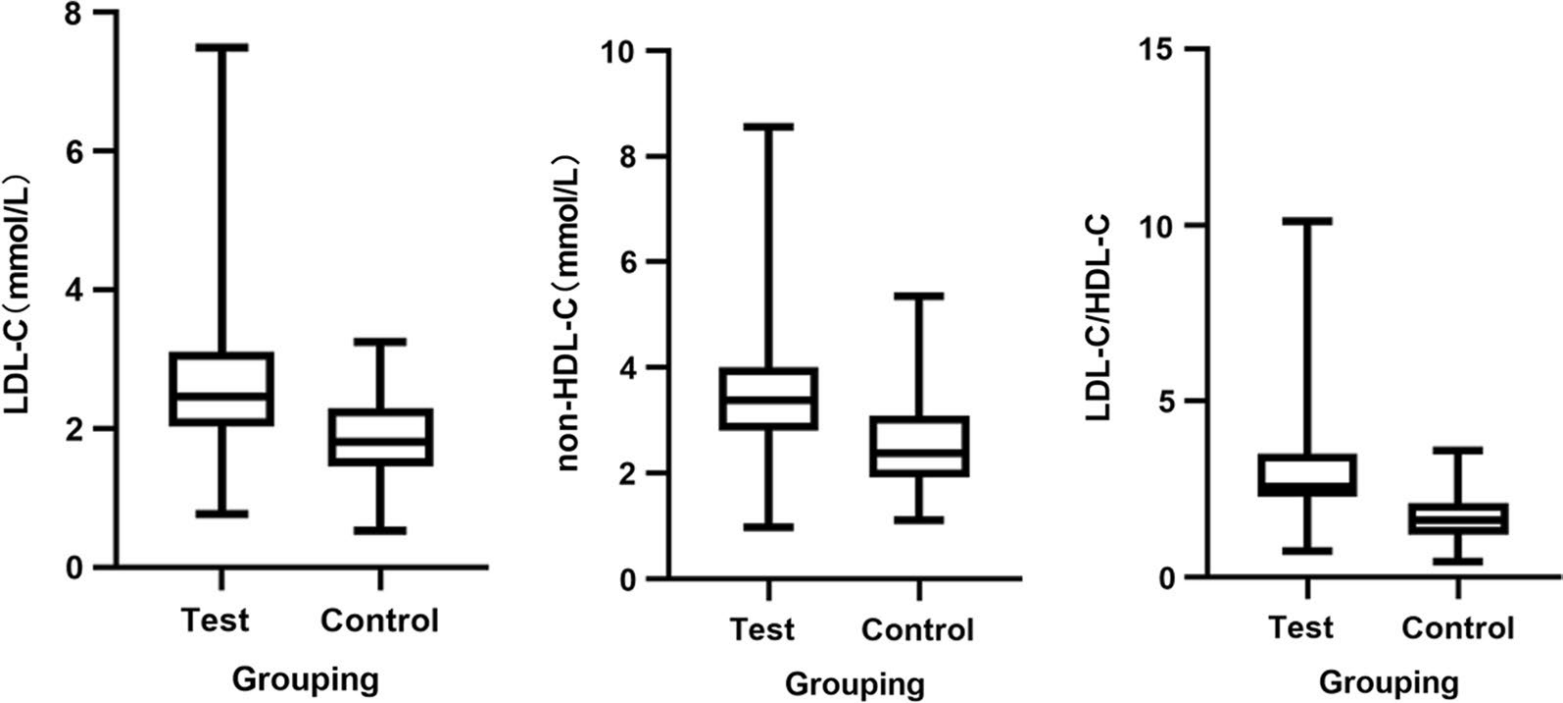
Data characteristics of LDL-C, non-HDL-C, and LDL-C/HDL-C in the two patient groups

In order to further clarify the above three indicators as risk factors for STEMI, the team performed a binary logistic regression analysis. Taking the control group as the low Gensini score group and the test group as the high Gensini score group, with LDL-C/HDL-C, non-HDL-C, and LDL-C as independent variables and Gensini score as the dependent variable, binary logistic regression analysis shows that LDL-C/HDL-C is an independent risk factor for STEMI (*OR* = 1.538, 95% *Cl*: 1.117 ~ 2.119, *p* < 0.05), further validating LDL-C/HDL-C^’^s effectiveness as an index predictor of STEMI. (Table 4, Fig. 3).

**Table 4:** Binary multi-factor logistic analysis of LDL-C, non-HDL-C, and LDL-C/HDL-C with Gensini score

Variables	*β*	*SE*	*Wald*	*OR*	95% *Cl*	*p*
LDL-C (mmol/L)	0.018	0.286	0.004	1.018	0.581–1.783	0.950
Non-HDL-C (mmol/L)	0.323	0.191	2.857	1.381	0.950–2.007	0.091
LDL-C/HDL-C	2.429	0.284	73.166	11.351	6.506–19.805	0.000

## Discussion

High serum concentration of LDL-C is an important risk factor for CHD. Previous studies have shown that oxidatively modified low-density lipoprotein cholesterol (ox-LDL-C) plays a key role in the pathogenesis of atherosclerosis [6]. ox-LDL-C has a low affinity for the scavenger receptors of macrophages. As it enters blood circulation, it stimulates the release of adhesion molecules and chemokines, which are then absorbed by macrophages through scavenger receptors, leading to the formation of foam cells. This reaction leads to development and progression of coronary atherosclerosis [7].

The role of HDL-C in CHD is relatively clear. Studies have shown that low levels of HDL-C increase the risk of CHD [8]. The incidence of coronary heart disease doubles in people with HDL-C levels lower than 40 mg/dL. Even with LDL-C under 70 mg/dL or TC under 200 mg/dL, patients with low HDL-C still have a significant risk of CHD [9]. Patients with low levels of LDL-C and HDL-C are still at high risk of atherosclerosis. At present, LDL-C is mainly used clinically to predict the risk of arteriosclerotic cardiovascular disease. However, though clinical intervention can help patients’ LDL-C level reach the recommended 1.4 mmol/L, some will still develop arteriosclerosis. Clinicians still seek better indicators to control the residual risk of ASCVD [5], so the ratios of AI, non-HDL-C, and LDL-C/HDL-C in the blood lipid profile have attracted attention. Changes in these ratios are proven indicators for evaluating CHD risk [10, 11].

Previous studies show that AI and low-density lipoprotein cholesterol (sd-LDL-C) have a significant negative correlation; moreover, sd-LDL-C is strongly related to coronary artery spasm, angina pectoris, and coronary artery stenosis [12]. Recently, an increasing number of analyses have shown that AI is related not only to atherosclerosis, but also to insulin resistance. In 2001, the United States National Cholesterol Education Program (NCEP) first evaluated non-HDL-C as a concept [13]. The latest 2019 European Society of Cardiology/European Society of Arteriosclerosis guidelines further adjusted the target values of LDL-C and non-HDL-C. For patients with extremely high cardiovascular risk, the recommended primary goal is LDL-C < 1.4 mmol/L (or LDL-C reduction > 50%), and the recommended secondary goal is non-HDL-C < 2.2 mmol/L [5].

There is a positive linear correlation between LDL-C/HDL-C ratio and changes in coronary plaque volume [14]. Vulnerable plaque is the main cause of CHD. Increased ratio of LDL-C/HDL-C is a predictor of coronary artery lipid-rich plaque and plaque vulnerability, conditions which can lead to an increased risk of sudden death [15, 16]. Coronary thrombosis is mainly caused by rupture of high-risk vulnerable plaques, which ultimately lead to acute myocardial infarction. The ratio of LDL-C/HDL-C can also inform lipid-lowering therapy; additionally, it is an evaluation index for prognosis of CHD patients [17]. Data from clinical trials [18] associates high LDL-C/HDL-C ratio with plaque progression in the coronary arteries. The reduction of the LDL-C/HDL-C ratio through drug intervention may be related to the reduction of coronary plaque [19].

Studies evaluating the above two indicators, though high in quantity, mainly examine the larger scope of coronary heart disease. This study, on the other hand, explores whether the above two indicators have an evaluative effect on the severity of coronary artery disease in STEMI patients, identifies which of the two can better gauge the severity of coronary artery disease in STEMI patients, and determines the boundary value that requires intervention.

Hypertension, hyperglycemia, high LDL-C, and smoking are very clear risk factors for atherosclerosis. But even after controlling these risk factors, some patients still experience cardiovascular events. These remaining causes are residual risks for cardiovascular events. The levels of HDL-C and triglyceride in the blood lipid profile are important residual risks [20].

In our study, indexes such as BMI, diabetes history, history of hypertension, smoking habits, ANC, TLC, TC, TG, HDL-C, LDL-C, VLDL-C, non-HDL-C, AI, and LDL-C/ HDL-C were significantly different in the two groups of patients (*p* < 0.05), demonstrating that the above indicators are high-risk factors for STEMI, a finding that is consistent with previous studies. The TC, TG, LDL-C, HDL-C, non-HDL-C, AI, and LDL-C/HDL-C of STEMI patients correlated with the patient’s Gensini score.

Because smoking, hypertension, and diabetes greatly impact the progression of coronary heart disease, we used those three factors, along with the above seven indicators, as independent variables, and Gensini score as the dependent variable. Stepwise multiple linear regression analysis found that the two indicators of LDL-C/HDL-C and non-HDL-C had a positive correlation with the Gensini score(*R*^2^ = 0.423, *β* = 0.518, *β* = 0.150, *p* < 0.05), with LDL-C/HDL-C being the most relevant. Even after treating other risk factors like smoking, hypertension, and diabetes, LDL-C/HDL-C and Gensini score still have significant correlation with severity of coronary artery disease in STEMI patients. Further analysis found that LDL-C/HDL-C can accurately predict whether patients with chest pain are STEMI (AUC: 0.880, 95% *Cl*: 0.847–0.912; *p* < 0.05). When the cut-off value is 2.15, the sensitivity is 0.845 and the specificity is 0.202. This is a high sensitivity. When the ratio of LDL-C/HDL-C is greater than 2.15, patients with chest pain are more likely to have STEMI.

### Study strength and limitations

Our results provide evidence that LDL-C/HDL-C assesses the severity of coronary artery disease in STEMI patients better than traditional LDL-C, making it a critical value that needs to be controlled. This study is a single-center retrospective study with a small sample size and fewer research indicators included. The rate of patients lost through lack of follow-up was high, and no good longitudinal study was carried out.

## Conclusion

We found that LDL-C/HDL-C works more effectively than non-HDL-C not only in evaluating the severity of coronary artery disease in STEMI patients, but also in predicting whether patients with chest pain are STEMI.

Current diagnosis and treatment guidelines recommend that patients' serum LDL-C levels be used to assess the risk of ASCVD. However, as mentioned above, even patients with well-controlled LDL-C are at risk of ASCVD. LDL-C’s propensity for causing cardiovascular disease has earned it the nickname “bad cholesterol,” while HDL-C, which has a protective effect on the cardiovascular system, is often called “good cholesterol.” When the patient's serum LDL-C and HDL-C levels are both low, the patient is at risk of cardiovascular disease. Using exclusively LDL-C to assess patients’ risk of cardiovascular disease has its limitations; non-HDL-C and LDL-C/HDL-C combined can more comprehensively assess the risk of cardiovascular disease than LDL-C alone.

We found that LDL-C/HDL-C is more related to the severity of coronary artery disease in STEMI patients than LDL-C and non-HDL-C. The lower the serum LDL-C/HDL-C level, the lower the risk of STEMI. In clinical diagnosis and treatment, when using traditional LDL-C to assess the patient’s lipid control, we should also consider both LDL-C and HDL-C, thus reducing residual risks.

## Supplementary Information

**Additional file 1.** Research data.

## Data Availability

All data generated or analyzed during this study are included in this published article and its Additional file 1.
